# Microbial fuel cells to monitor natural attenuation around groundwater plumes

**DOI:** 10.1007/s11356-024-35848-5

**Published:** 2025-01-04

**Authors:** Panagiotis Kirmizakis, Mark Cunningham, Deepak Kumaresan, Rory Doherty

**Affiliations:** 1 Center for Integrative Petroleum Research, College of Petroleum Engineering and Geosciences, King Fahd University of Petroleum and Minerals, Dhahran, Saudi Arabia; 2 School of Biological Sciences, Queen’s University Belfast, Belfast, Northern Ireland BT7 1NN UK; 3 School of Natural and Built Environment, Queen’s University Belfast, Belfast, Northern Ireland BT7 1NN UK

**Keywords:** Microbial fuel cell (MFC), Monitored natural attenuation (MNA), Carbureted water gas (CWG)

## Abstract

This research presents a straightforward and economically efficient design for a microbial fuel cell (MFC) that can be conveniently integrated into a borehole to monitor natural attenuation in groundwater. The design employs conventional, transparent, and reusable PVC bailers with graphite tape and granular activated carbon to create high surface area electrodes. These electrodes are connected across redox environments in nested boreholes through a wire and variable resistor setup. The amended electrodes were installed in pre-existing boreholes surrounding a groundwater plume near a former gasworks facility. Among all the MFC locations tested, the MFC at the plume fringe exhibited the highest electrical response and displayed significant variations in the differential abundance of key bacterial and archaeal taxa between the anode and cathode electrodes. The other MFC configurations in the plume center and uncontaminated groundwater showed little to no electrical response, suggesting minimal microbial activity. This straightforward approach enables informed decision-making regarding effectively monitoring, enhancing, or designing degradation strategies for groundwater plumes. It offers a valuable tool for understanding and managing contaminant degradation in such environments.

**Supplementary Information:**

The online version contains supplementary material available at 10.1007/s11356-024-35848-5.

## Introduction

Monitored natural attenuation (MNA) of contaminant groundwater plumes is a favored remediation approach for managing potential threats to the natural ecosystem and the well-being of individuals (Jørgensen et al. 2010; Newell et al. 2021). MNA relies on the subsurface’s natural biological, chemical, and physical processes to reduce contaminants’ quantity, concentration, flux, or harmfulness (Carey et al. 2000). These processes include biodegradation, sorption, and dilution, which work together to control, reduce, or maintain the plume at a steady state. Effective monitoring of these mechanisms is crucial to ensure proper plume management.

However, monitoring biological activity to ensure biodegradation can be challenging and costly (Anneser et al. 2010). In heavily contaminated plumes with excess electron donors, biodegradation often occurs at the plume fringe, where electron acceptors are resupplied by surrounding uncontaminated groundwater (Breukelen and Griffioen 2004; Prommer et al. 2006; Tuxen et al. 2006). Recent research challenges our comprehension of the spatial mechanisms of natural attenuation (NA) processes, especially biodegradation around contamination plumes (Meckenstock et al. 2015). Comprehensive soil and groundwater sampling is frequently required to discern these mechanisms, yet it may not always be practical in routine site investigations and monitoring. As a result, remedial verification and monitoring are usually time-consuming and expensive, particularly for NA, which requires extensive microbial analysis to understand ongoing metabolic processes.

Developing innovative sensor technologies for bioremediation or NA systems is essential to address these challenges. Conceptual models and verification of microbial activity occupying ecological niches in plume fringes are needed. Microbial fuel cells (MFCs) have emerged, as promising tool, leveraging microbes’ ability to transfer electrons to solid electron acceptors like graphite (Gregory et al. 2004). MFCs have diverse applications, from power generation to medical devices, but the core concept involves microbes exchanging electrons for their metabolic gain with electrode surfaces. This process, known as bacterial extracellular electron transfer (EET), is vital in numerous biogeochemical cycles and processes of degradation (Chen et al. 2021; Ustra et al. 2021; Verma et al. 2023). Electrogenic microorganisms, capable of transferring electrons through EET, are extensively studied and utilized in engineered devices such as microbial fuel cells (MFCs) or bio-electrochemical systems (BESs). However, large-scale applications of MFC technology for remediation are not yet practical (Lu et al. 2014); designs of popular remediation systems that “retrofit” MFC technology have been proposed (Doherty et al. 2022).

Most MFC studies are lab-scale, using synthetic or low concentration feedstock, which limits their industrial applicability (Cecconet et al. 2020). Field-scale MFC studies typically use short, non-continuous electrodes and focus on active remediation methods, such as substrate injection and current amendments (Williams et al. 2010). These approaches often overlook the complexity of microbial communities involved in natural attenuation, and ongoing modifications may not be feasible over an extended period. Moreover, native microbial populations capable of direct or indirect electron transfer already exist in groundwater, eliminating the need for exogenous bacteria (Kirmizakis et al. 2019).

Studies of microbial diversity associated with contaminated groundwater plumes at gasworks sites indicate variations due to contaminant concentration and toxicity (Ferguson et al. 2007, 2003). Enhanced microbial activity is observed at the plume fringe, where oxygen-depleted plume meet surrounding groundwater (Winderl et al. 2008). Biogeophysical studies detect self-potential differences at the edges of gasworks plumes, known as bio-geobatteries, which may indicate NA processes (Heenan et al. 2017; Kirmizakis 2020). These signals arise from the sharp electrochemical gradients between the plume and clean groundwater, highlighting microbial activity. Such biogeochemical processes can be monitored in situ using geo-electrical techniques such as resistivity, induced polarization, and self-potential (Kirmizakis et al. 2023, 2015; Nyquist and Corry 2002).

MFCs present an innovative and highly adaptable approach for monitoring microbial activity in situ, mainly when applied in field-scale studies. The ability of MFCs to capture real-time microbial processes through EET provides unique insights into NA processes. Despite the inherent heterogeneity of subsurface environments, MFCs can still detect key microbial activities, particularly at plume fringes where biological processes are often most active. While subsurface variability can influence spatial resolution, the strategic deployment of MFCs in critical locations such as plume cores, fringes, and uncontaminated zones enables a more targeted and focused monitoring approach. By coupling MFCs with complementary techniques like resistivity or groundwater sampling, the spatial coverage and resolution can be further enhanced, making this technology a promising tool for field-scale applications. These advantages position MFCs as an effective, real-time monitoring system that can streamline the assessment of microbial activity even in complex subsurface environments (Kumari et al. 2022).

This study will apply passive field-scale MFCs in boreholes at a groundwater plume fringe, plume core, and non-contaminated area. The aim is to examine whether the electrical output from an MFC can serve as a simple indicator for microbial activity associated with NA in and around contaminated plumes rather than the specific remediation of contaminants.

## Preliminary site investigation

### Land-use and geology

The investigation site, located at a former gas production facility in Northern Ireland, was operational for over 150 years. During its operational period, by-products of coal gasification led to significant contamination of soil and groundwater, posing substantial risks to human health and the environment. In the late 1980s, following the plant’s closure, remediation and redevelopment efforts were undertaken. However, these efforts primarily targeted the shallow subsurface, leaving deeper contamination unaddressed. Contaminants, including those in the groundwater, persist at depths exceeding 12 m below the surface. The site is underlain by a range of geological formations, including recent estuarine alluvium, glacial-fluvial sands, glacial till (boulder clay), and Triassic Sandstone bedrock. Across all boreholes drilled on the site, anthropogenic deposits were encountered, with thicknesses varying between 1.0 and 10.9 m. These deposits consist of gray gravelly, sandy, silty clay, bricks, concrete, timber, intermittent cobbles, patches of cinder, and sporadic slag deposits. Groundwater in this unit generally flows in from south-southwest to north-northeast direction, following the meanderings of the nearby river Lagan.

Beneath the anthropogenic layer, glacial deposits are commonly observed, consisting of brown, slightly sandy, silty clay interspersed with thin sandy lenses, ranging from orange to reddish-brown in color, and typically of fine to medium grain size. The underlying Triassic Sandstone exhibits transmissivity values between 11 and 39 m^2^/day, with hydraulic conductivities ranging from 0.01 to 0.2 m/day. Groundwater in this bedrock unit flows in a southwest to northeast direction, ultimately discharging into Belfast Lough (Burns 2013).

Historical site data, along with identified potentially polluting activities, point to the primary source areas of contamination being associated with the gasholder bases. Although there is no official documentation of leaks prior to the site’s redevelopment, it was a common practice in the manufactured gas plant industry to dispose of waste materials, such as tars, on-site production areas (Murphy et al. 2005). Contaminants associated with these sources include coal tars and oils, ammonia liquor, and coal dust. Additionally, during the demolition of historical gasholders, tarry sludges were often treated as informal landfills, backfilled, and subsequently forgotten about (Thomas 2014). This historical context, combined with the persistence of deep-seated contaminants, highlights the need for further investigation and remediation efforts to fully address the environmental risks posed by this former industrial site.

### Conceptual model of the subsurface environment

To ensure the effective emplacement of MFCs, it was crucial to first establish a conceptual understanding of the contaminant hydrogeology at the site, identifying potential migration pathways and areas of concern. Groundwater levels and chemical parameters were analyzed from samples collected at existing boreholes across the site (Table 1). The groundwater chemistry reflected typical characteristics of manufactured gas plant sites, including elevated levels of total organic carbon, consistent with findings from similar studies (Gallacher et al. 2017).

**Table 1:** Water quality results of overburden and sandstone aquifers

Well ID*	Piez. depth (m)	Temp. (°C)	pH	ORP (mV)	Cond. (µS/cm)	Dissolved oxygen	Ammonical nitrogen (mg/l)	Manganese (µg/l)	Sulfate (mg/l)
Overburden (unconfined) aquifer									
B01	3.646	15.5	6.91	− 274	987	0.36	0.93	210	83
B02	3.529	14.34	7	− 230	1238	0	7.8	720	900
B04	7.22	16.58	7.14	− 294	771	0.08	3.6	230	240
B05	3.052	16.04	6.55	− 278	949	0.91	11	240	72
B06	2.589	12.97	7.93	− 213	645	3.81	N/A	N/A	N/A
B07	3.382	1.53	6.59	− 252	855	0.22	1.4	680	24
B09	8.442	12.74	9.52	− 318	671	0.52	16	14	61
BH101	4.073	11.85	7.23	− 137	838	2.17	0.93	260	110
WS200	6.206	13.32	7.03	− 198	936	2.40	0.41	5.5	21
WS202	4.773	12.79	7.19	− 190	3277	1.47	3.5	290	690
WS204	4.443	11.86	6.69	− 156	3557	1.57	N/A	N/A	N/A
WS206	5.39	10.49	8.18	− 232.2	3619	1.48	4.4	44	280
Sandstone (confined) aquifer									
SS1	19.80	12.87	6.96	− 282	875	0.0	0.11	880	100
SS2	23.15	11.9	7.17	− 234	705	0.0	0.31	16	82
SS4	16.5	13.28	8.4	− 367	891	0.0	32	30	350

^*^Well IDs as documented by Belfast City Council

**Fig. 1:**
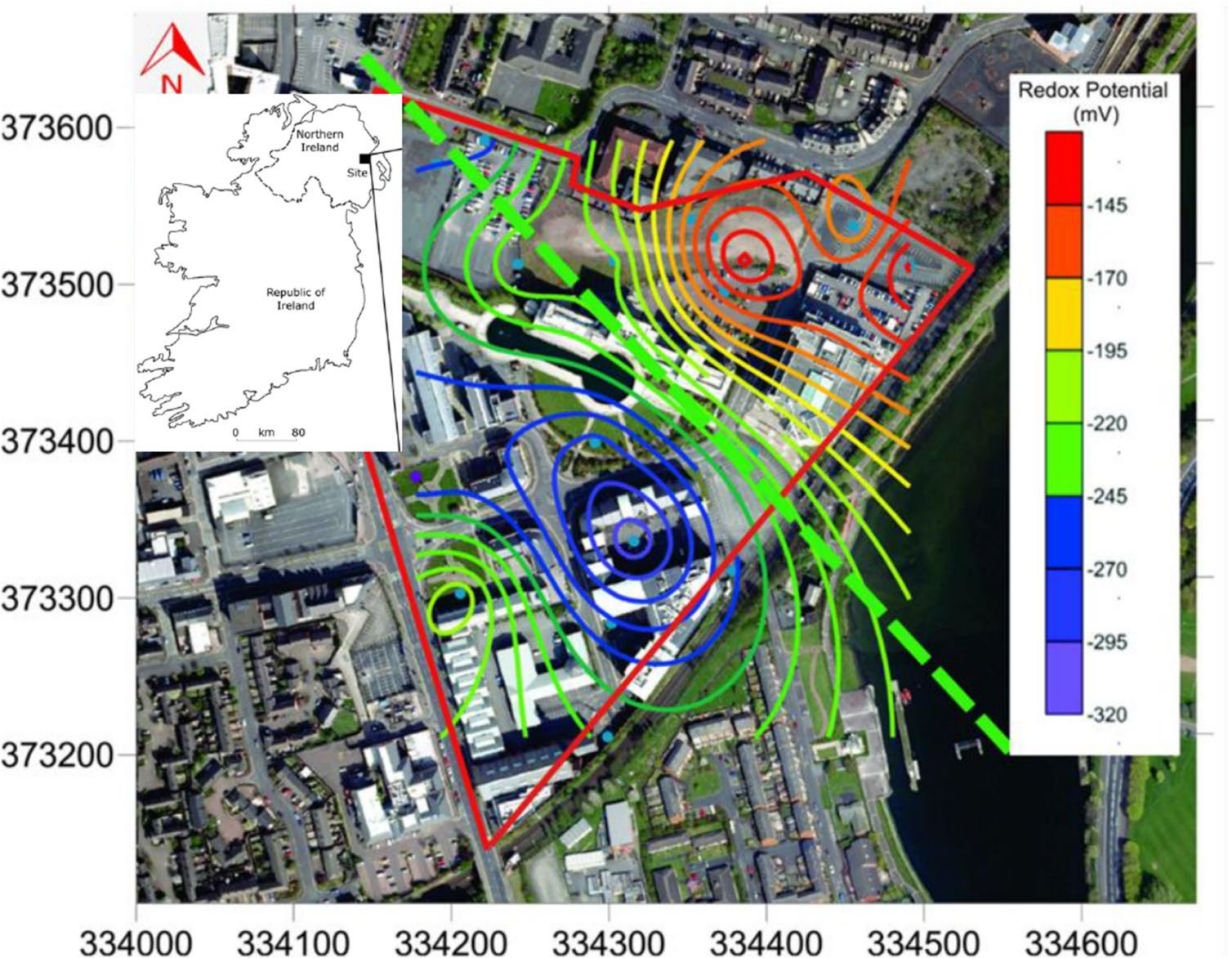
Groundwater oxidation–reduction potential (ORP) of overburden aquifer averages − 226 mV. The minimum value is − 137 mV and the maximum − 318 mV

To assess the redox conditions within the overlying aquifer, oxidation–reduction potential (ORP) measurements were conducted using a TPS 90FL-T multi-parameter field logger, as depicted in Fig. 1. The results indicated a predominantly reducing environment, with a median redox potential of − 226 mV. Notably, the northern section of the site exhibited mildly reducing conditions, with an ORP of approximately − 100 mV, whereas the southern section showed significantly lower ORP values at around − 300 mV, indicating the presence of an anaerobic microbial environment. This region corresponds to the main body of the contaminant plume (Søndergaard 2009).

Post-remediation monitoring revealed high conductivity in shallow groundwater samples, which is attributed to residual surface contamination. Additionally, the analysis of ionic species, heavy metals, and hydrocarbons revealed heterogeneity in their distribution within the overlying aquifer, with distinct localized patterns.

The groundwater samples from the overburden aquifer exhibited low concentrations of BTEX (benzene, toluene, ethylbenzene, and xylene) compounds, with minor variability between individual compounds. However, elevated concentrations were observed in deeper samples from the underlying sandstone aquifer. Weathering mechanisms appeared to influence the distribution of BTEX compounds, with varying degrees of retardation for each compound. As outlined in Supplementary Table S1, the order of retardation was observed as E > X > T > B, with ethylbenzene (E) and xylene (X) showing similar geochemical behavior. Retardation factors (R) for the overburden and sandstone aquifers were calculated using a linear equilibrium sorption model. Benzene (B) and toluene (T), being more soluble among the BTEX compounds, tend to decrease R1 and R3 close to the primary contamination source, either due to dilution or transport. Nonetheless, biodegradation can also impact these parameters within the groundwater near the contamination source, particularly in anaerobic conditions, adding complexity to the interpretation of the findings (Alvarez et al. 1998).

Under anaerobic conditions, benzene degrades more slowly compared to other BTEX compounds. As benzene degrades, it consumes dissolved oxygen, shifting the local environment to anaerobic conditions. Consequently, benzene degraders are primarily found at the plume’s fringes, where oxygen is more readily replenished from surrounding groundwater. The retardation factors decrease near the plume due to dilution and transport, while biodegradation can either increase or decrease these factors depending on the mechanisms at play. Elevated values observed in wells B05, B07, and WS206, located farther from the plume center, are likely the result of dilution and biodegradation. In contrast, well SS2, in the sandstone aquifer, exhibited high retardation factors, suggesting enhanced biodegradation processes. Well SS4, located closer to the plume center but still removed from the primary source, also showed evidence of degradation, indicating that active natural attenuation processes are occurring.

Based on the distribution of contamination and degradation patterns, the region between wells SS4 and SS2 appears to be an optimal location for the installation of MFCs along the plume fringe, with well SS4 serving as the plume center for MFC placement. Additionally, well SS1, which exhibited minimal contamination, is identified as a suitable candidate for a clean control MFC. However, microbial analysis revealed a consortium indicative of previous contaminative activity at this location (Costeira et al. 2019), suggesting that contaminants may have been present historically and have since undergone biodegradation, further evidence of ongoing NA processes at this site.

In conclusion, the southern section of the site shows significant groundwater contamination at depth, with clear evidence of anaerobic degradation processes, while the northern section remains relatively uncontaminated, with aerobic conditions in the sandstone aquifer. These findings suggest that a large-scale MFC could be employed as a biosensor along the southern plume edge, with a control MFC positioned in the clean groundwater at the northern end. Additionally, an MFC placed near the center of the plume on the southern side would provide valuable insights into the behavior of the contaminant plume. This setup will allow for comprehensive monitoring of MFC performance across varying contaminant conditions, enhancing our understanding of the dynamics of the plume and the effectiveness of the MFC technology in such environments.

## Materials and methods

### MFC design and construction

**Fig. 2:**
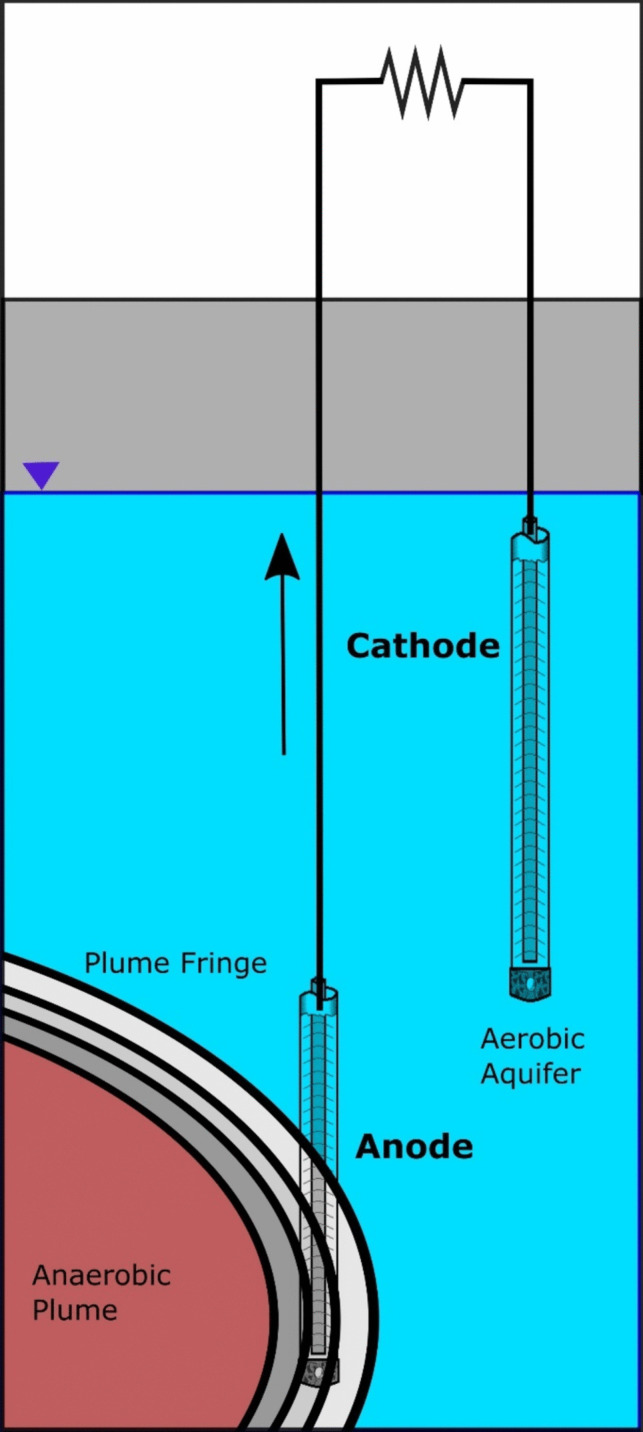
Microbial fuel cell design and operation. The anode electrode of the MFC is placed at the plume fringe where there is maximum microbial activity; the cathode electrode of the MFC is placed in a clean aerobic section of the same well. The anodes are connected via a wire and electrical properties are monitored

The MFC consisted of two large electrodes (~ 1m) positioned at different depths within nested piezometers. The contaminated deeper sandstone borehole served as the anode, while the shallower overburden borehole acted as the cathode of the MFC. The electrodes, made from PVC bailers filled with granular activated carbon (GAC) obtained from CarboTech AC GmbH and graphite tape (Fig. 2, Supplementary Fig. S1), were connected to a Madgetech Volt101A voltmeter for data-logging and a variable resistor box to monitor electrical output. GAC was chosen for its high conductivity and surface area, facilitating the growth of microbial communities that encompass both degraders and electrogenic species. Three MFCs were installed across the gasworks site, strategically positioned at the control/clean area (MFC 1), the plume center (MFC 2), and close to the plume fringe (MFC 3).

### Electrical monitoring

After installing the anode and cathode electrodes, a waiting period of several days was observed to allow the electrodes to stabilize before commencing voltage monitoring. This crucial step ensured that the charge was balanced and the electrical circuit was fully established (Elgrishi et al. 2018; Feng and Song 2016). The output current was calculated using Ohm’s law (I = V/R), with current (I) measured in amperes, voltage (V) in volts, and resistance (R) in ohms. A consistent resistor load of 10 kiloohms (10 kOhms) was maintained throughout the study. It is worth noting that laboratory studies often employ lower resistor loads, such as 1 kOhm or even less (Koók et al. 2021). However, in large-scale bioelectrochemical systems implementations, substantial potential reductions happen because of increases in ohmic resistance resulting from enlarged electrode dimensions, increased surface area, and the inherent resistance of wiring and connections (Kirmizakis et al. 2019; Rozendal et al. 2008). In such field applications, non-ohmic behaviors may arise due to these subsurface complexities, as well as fluctuations in microbial activity and contamination levels, which can lead to non-linear electrical responses affecting current accuracy. To address these complexities, polarization curves were generated by introducing periodic variations in the external load on specific days. The external load underwent exponential decreases from 10 megaohms (10 MOhms) to 10 Ohms, followed by a return to the higher load level every 2 min. This methodology provided a broader evaluation of MFC performance under varying external load conditions, though no further alternative methods were considered.

### Determination of bacterial and archaeal diversity using amplicon sequencing

After completing electrical monitoring, the MFC electrodes were removed and taken to the lab for sampling; three samples R1–R3 were taken from each electrode. We followed a standardized protocol for DNA extraction, library preparation, and sequencing across all samples to minimize variability due to sample preparation. Each step was conducted with strict adherence to quality control measures to ensure consistency, reducing the potential for bias introduced by handling differences. The MFC samples were soaked in 120 µL of 50% (v/v) acetone and incubated for 10 min at 50 °C with gentle shaking at 300 rpm using an Eppendorf ThermoMixer. After incubation, sterile swabs were used to absorb the acetone, and then dried under vacuum. Once almost dry, the swabs were placed into C1 solution, and DNA extraction was carried out using the Powersoil DNA extraction kit following the manufacturer’s instructions. The extracted DNA was eluted in 50 µL of sterile MQ water and quantified using the dsDNA system Quantifluor kit by Promega. Quality control (260/280 nm; 260/230 nm values) was assessed using a NanoDrop spectrophotometer. Samples with sufficient concentration were used for PCR amplification of the 16S rRNA gene (V4 region) using the primer set 515F (5’-GTGYCAGCMGCCGCGGTAA-3′) and 806R (5’-GGACTACNVGGGTWTCTAAT-3′. We chose a primer based on the V4 region of the 16S rRNA gene due to its wide taxonomic coverage, efficiency with high-throughput sequencing, and robust reference databases. It can potentially overlook functionally specialized microbes and excluding non-bacterial components of the community (Na et al. 2023), but for harsh environments, V4 has been shown to perform well (Fadeev et al. 2021). The PCR master mix was prepared according to the DreamTaq manual, with each 25 µL PCR containing 2.5 µL Dream buffer, 0.2 µM 515F primer, 0.2 µM 806R primer, 0.2 mM dNTPs, 0.2µL of DreamTaq polymerase, 3 µL (> 2 ng) of DNA template, and nuclease-free water from Sigma-Aldrich. PCR thermocycler conditions included an initial heating step at 94 °C for 3 min, followed by 30 cycles of denaturation at 94 °C for 45 s, annealing at 55 °C for 1 min, elongation at 72 °C for 1 min and 30 s, and a final elongation step of 10 min at 72 °C. DNA gel electrophoresis was performed for all PCR samples using 1% agarose and SYBR™ Safe DNA Gel Stain by Invitrogen. All PCR amplifications were performed in triplicate, pooled, purified using the AMPure beads protocol, and sent for amplicon sequencing at the Core Technology Unit Genomics (Queen’s University Belfast) using MiSeq platform. The raw 16S rRNA gene sequences are available at the European National Archive (ENA) under accession ID PRJEB64905. Bioinformatics analysis was performed using the QIIME2 platform (v2019.4) (Bolyen et al. 2019). We also used stringent quality filtering and trimming steps within the DADA2 pipeline in QIIME2 to remove low-quality sequences, ensuring that retained sequences represented true biological diversity. This further minimized potential biases introduced by sequencing errors. Quality control analysis and trimming were conducted with DADA2 (v2019.4.0) (Callahan et al. 2016), applying a quality score of 25 and truncating sequences at 240 bp. Primers were removed by trimming the first 19 bases of the forward sequences and trimming off the first 20 bases of the reverse sequences. Sequences were aligned using MAFFT (Katoh and Standley 2013), and a phylogenetic tree was built with FastTree2 (Price et al. 2010). Taxonomy was assigned to sequences using QIIME feature-classify (Bokulich et al. 2018) with classify-sklearn (Pedregosa et al. 2011) using a pre-trained classifier generated from the SILVA (Quast et al. 2013) databases available in QIIME2 resources in 2019 (Silva 132 99% OTUs from 515F/806R region of sequences). The QIIME2 data was exported as a table with taxonomy (V2) in BIOM format along with the Newick phylogenetic tree and taxonomy BIOM file. We assessed and accounted for differences in sequencing depth by rarefying the samples to a uniform depth, which is a common approach in microbiome studies to control for variability in sequencing effort across samples. Additionally, we included alpha diversity metrics after rarefaction to verify that sample richness was adequately captured at the chosen sequencing depth. The sequences were imported into R for filtering and statistical analysis using the phyloseq pipeline (McMurdie and Holmes 2013); a p-sampling depth of 6784 is applied based on rarefaction curve analysis (Supplementary Fig. S3). Unassigned sequences at a kingdom/domain and at phylum level were removed. Differential abundance analysis was performed using DESeq2 (Michael I Love et al. 2014a, b) (alpha = 0.05) to compare taxonomic relative abundance differences of bacterial 16S rRNA sequences agglomerated to genus level. A scaled heatmap is constructed based on the differentia abundance data using the ComplexHeatmap (version 2.18.0).

### Chemical analysis

Groundwater analysis incorporated the utilization of 2D gas chromatography (GC) with a flame ionization detector (GC × GC FID) to identify total petroleum hydrocarbons (TPH) and GC with mass spectrometric detector (GC–MS) to identify semi-volatile organic compounds. Both GC × GC FID and GC–MS represent robust techniques frequently applied in environmental analysis and studies on contaminant transformation (Arey et al. 2022; Chibwe et al. 2017; Mao et al. 2009; Moore et al. 2021). GC × GC, which divides the sample into two GC columns, offers valuable insights into the sample’s retention time and polarity with a single injection, thus streamlining the analysis process (Welke and Zini 2011). The samples were taken using a Watson-Marlow peristaltic pump and kept chilled until transported to an MCERTs-accredited laboratory Chemtest, Newmarket, UK. The sampling process for this analysis was designed to avoid contamination, incorporating the extraction of an extensive quantity of groundwater through pre-installed pipes to ensure consistent access. This controlled approach minimized the risk of introducing additional contaminants, including during the purging process, ensuring that the results accurately reflected in situ conditions rather than any artifacts of the sampling method.

### Surface area analysis

Following the 131-day monitoring period, the GAC from each electrode was sampled, air-dried, and subsequently stored. The surface area of both the original GAC and the samples retrieved was determined using a gas adsorption analyzer (TriStar II 3020 Micrometrics). To assess any structural changes in the GAC due to precipitation processes, X-ray diffraction (XRD) analysis was conducted on all samples, including the untreated initial GAC. The XRD analysis was carried out in a PANalytical X'Pert PRO MDP diffractometer, with all samples finely ground and thoroughly homogenized prior to analysis.

## Results

### Electrical monitoring

One of the main hypotheses of the field experiment was that the MFC placed at the plume fringe would generate greater electrical output due to the reducing, electron-rich environment created by the anaerobic degradation of organic contaminants. This process would transfer excess electrons via a wire to the installed cathode, leading to a significantly higher electrical response compared to the other two locations, which were expected to yield lower electrical outputs. The plume fringe MFC (MFC3) indeed exhibited remarkable performance, with a 194% higher electrical production (219.587 mV) than the control MFC (MFC2) (3.316 mV) and a 186% higher output than the center plume MFC (MFC1) (7.611 mV) during the operational period with a 10-kOhms resistor (Table 2).

**Table 2:** Average electrical output of MFCs on site during 10 kΩ resistor operations

	Average voltage (mV)	Average current (µA)
MFC1 (control)	3.316	1.35
MFC2 (plume center)	7.611	0.99
MFC3 (plume fringe)	219.587	21

Figure 2 presents a visualization of the impact of situating a microbial fuel cell (MFC) near the edge of the plume instead of its placement at the center. The graph depicts the recorded voltage changes over time, with MFC 3 exhibiting a notably stronger signal. Initially, a distinct lag phase was observed in all MFCs, which can be attributed to the adaptation of the microbial community. Subsequently, an exponential growth phase followed, leading to the establishment of a stable population phase. Interestingly, MFC 3 demonstrated a rapid escalation in electrical response after the pass of 20 days, indicating rapid colonization by degrading and electroactive microbes.

**Fig. 3:**
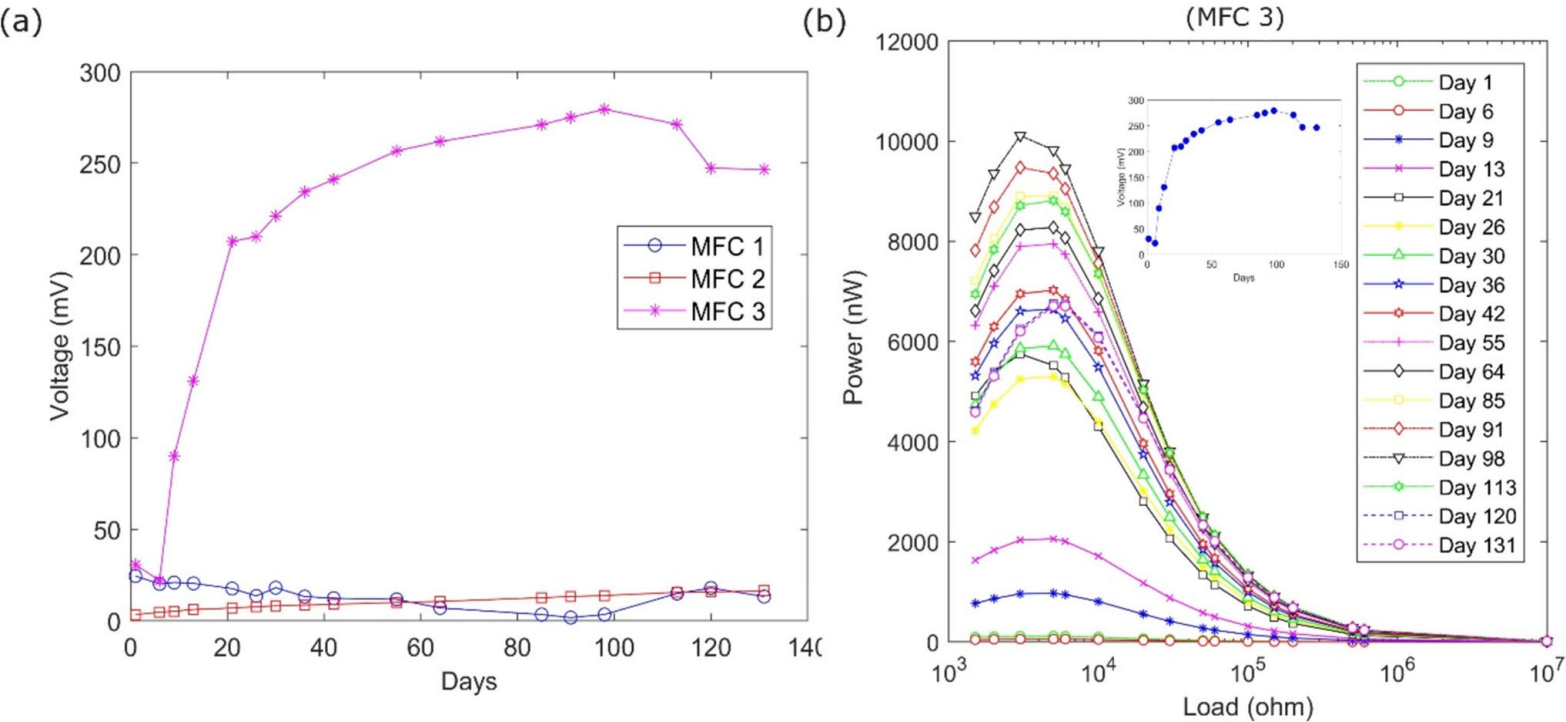
MFC output of **a** voltage profiles of bio-electrochemical systems installed on site during 131 days of monitoring. The selected points represent the measured voltage in 10 kΩ resistor operation. MFC3 is more effective in terms of voltage output, and **b** power curves of MFC3 across a wide range of resistances. There is a noticeable increase after 20 days of operation. Insert graph represents the current values of MFC3 with a resistance of 10 kΩ

The voltage profile of MFC 3 over time, as illustrated in Fig. 3a, exhibited a clear exponential growth from days 5–21 which stabilized over the 131 days of the experiment. During the monitoring period, MFC 1 (control) displayed a very low electrical response due to the lack of microbial growth on the electrodes. Initially, MFC 1 exhibited a high output upon installation, but it subsequently experienced a linear decline, in contrast to the other MFCs, which demonstrated increased signals over time. This decline in MFC 1’s output indicates rapid GAC saturation, leading to a significant drop in performance. The low electrical output can largely be attributed to the uncontaminated environment, which lacks sufficient electron donors to drive microbial extracellular electron transfer. While factors such as electrode placement and groundwater flow could contribute to some extent, the absence of a robust microbial community is the primary driver of the limited electrochemical activity observed. Slight electrical fluctuations (< 3 mV) were documented in the aftermath of rainfall events, which can be attributed to subsurface electrokinetic potential generated by the movement of electrolytic fluids through the porous media.

For MFC 2 in the plume center, the presence of high levels of contamination limited biodegradation, resulting in an inability to offer a signal that is both strong and consistent. This observation aligns with Kim et al. (2007), who noted the impact of contamination toxicity on microbial fuel cells. Elevated toxicity levels can impede metabolism and electron transfer mechanisms, resulting in a diminished electrical signal (Conners et al. 2022; Corbella et al. 2019; Shirkosh et al. 2022). The output from MFC 2 corroborates the idea of depletion of electron acceptors and the suppression of bacterial metabolic processes in the central region of the plume. These responses were indicative of a stable yet modest increase in the measured electrical output, which was overshadowed by the performance of MFC 3.

Figure 3b presents the calculated power and current (inert graph) output of MFC 3 over a spectrum of resistance settings during the measurements. MFC 3 consistently outperformed the other MFC units on the site in terms of both power and current output. This superior performance can be attributed to the heightened microbial biodegradative activity occurring at the plume’s periphery, which generates electrons through the anaerobic oxidation of organic contaminants. These electrons are then transferred through the MFC, resulting in the observed electrical output. The current output of MFC 3 exhibited a distinct lag phase within the initial week, which can be attributed to the adaptation of the microbial ecology to the electrodes. Subsequently, an exponential growth phase was observed between days 9 and 13, and again from day 21 onward, transitioning into a stable population that persisted until day 120, at this point, a potential decline phase appeared to initiate. On average, the current output of MFC 3 remained at 20 µA over the course of the 131-day operational period.

### Bacterial diversity

DNA was successfully recovered from the MFC 2 anode and cathode (plume center) and the MFC 3 anode and cathode (plume fringe). However, it was not possible to recover DNA from the control MFC 1. This outcome was expected since the control area had no contamination, and it would take a considerable amount of time to establish a microbial community on the MFC without a source of energy (biodegradable contaminants).

**Fig. 4:**
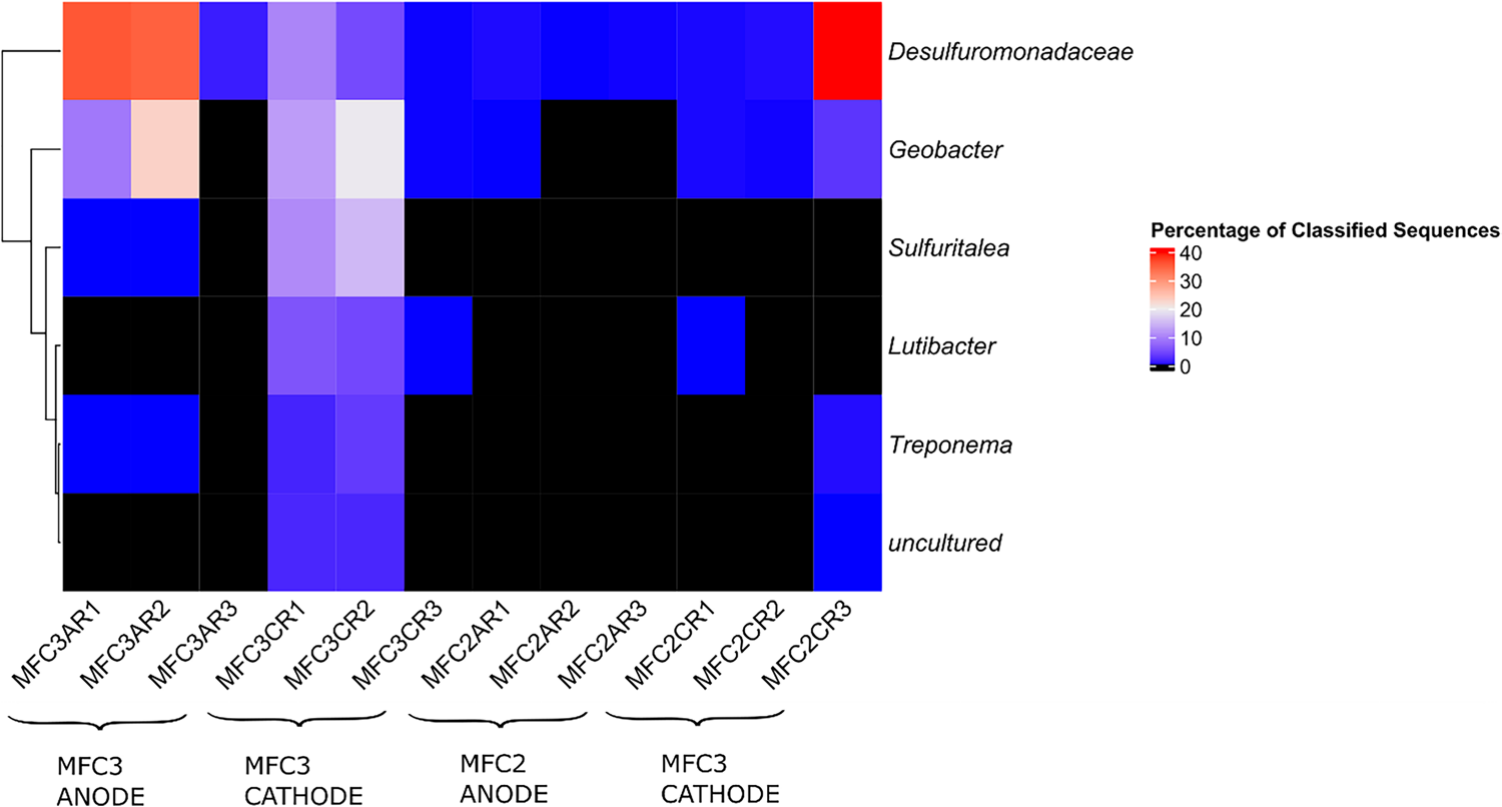
Differential abundance analysis performed using DESeq2 (Michael I Love et al. 2014a, b) (alpha = 0.05) to compare taxonomic relative abundance differences of bacterial 16S rRNA sequences agglomerated to genus level, highlighting microbiota differences in ecological niches at the plume fringe and plume center, as well as between the anode and cathode environments. MFC3 = high electrical output, MFC2 = low electrical output. Labelling notation MFC3AR1 = MFC3, anode, replicate 1

QIIME2 platform was used to classify and annotate the amplicon sequences and compare the relative abundance of 16S rRNA gene sequence data alongside beta diversity and rarefaction curves (Supplementary Fig. S2-4) and identified the relative abundance of dominant species found on MFC electrodes using the differential abundance analysis via DESeq2 tool (Fig. 4) (Love et al. 2014a, b). This analysis identified the key differences in the abundance of each taxa between two classes of subjects or samples and assigns a significance value (alpha = 0.05) to each comparison (Cappellato et al. 2022). We specifically investigated the differences in ecological niches at the plume fringe and plume center, as well as between the anode and cathode environments.

Samples from the anode and cathode at the plume fringe (MFC 3) revealed a higher relative abundance of key electrogenic taxa such as *Desulfuromonadaceae* and *Geobacter* which aligns with the electrical data showing a significantly higher output from MFC 3 at the plume fringe compared to MFC 2 in the plume center (Fig. 4, Supplementary Fig. S2).

Surprisingly, the differential abundance analysis heatmap shows that *Desulfuromonadaceae* is common in all samples. There was significant relative abundance in both anode and cathode samples in MFC3 (high electrical output) and in one sample from the cathode of MFC2 (low electrical output). They are an electrogen normally found within anodes in benthic microbial fuel cells and have been associated with cable bacteria (Reimers et al. 2017). *Geobacter* is an electrogen and degrader (Kronenberg et al. 2017) also had significant relative abundance in the anode and cathode of MFC3 (high electrical output) and to a lesser extent in the cathode of MFC2. MFC3 (high electrical output) also has greater relative abundance of Sulfuritalea especially within the cathode, which is capable of aerobic degradation of light aromatic compounds, typically found in gasworks contamination (Sperfeld et al. 2019). Additionally, sequences affiliated to the genus *Lutibacter* was also detected in the MFC 3 cathode and has been previously detected in both biocathodes (Philippon et al. 2021) and PAH-contaminated sediments (Isaac et al. 2013). Although the cathode boreholes are in the overburden above the plume, these are still prone to residual “hotspot” contamination that was missed by earlier remediation attempts. *Treponema* abundance was also found primarily on MFC3; they have been noted to occur in areas of PAH contamination as successors of microorganisms that degrade hydrocarbons (Júlio et al. 2019) suggesting active degradation of hydrocarbons at this location. The distribution of *Desulfuromonadaceae and Geobacter* across both anodes and cathodes suggests that there can be localized communities in large electrodes even within active MFCs. This could be due to large size (~ 1m in length) of the down borehole electrodes where localized ecological niches of microbial communities may occur.

### Chemical analysis

Table 3 shows the outcomes of the GC-FID analysis conducted on the collected samples both before and after system’s installation. The contamination primarily consisted of lighter aromatic fractions, which align with the characteristics of a carbureted water gas (CWG) process (Hauswirth 2014; Speight 2018; Stout and Brey 2019). Notably, an increase in ammonium degradation was observed, which can influence the aerobic degradation of organic contaminants through preferential oxygen consumption and establish the boundary between aerobic and anaerobic conditions at the plume’s periphery. Previous research conducted at gasworks sites has identified aerobic ammonium oxidizers like *Nitrospira* and members of phylum OD1 (Costeira et al. 2019). This suggests a microbial conversion of ammonia into nitrite and nitrate, contributing to maintaining a sharp electrochemical gradient at the plume edge and its stable condition.

**Table 3:** Aliphatic and aromatic hydrocarbon groundwater concentrations in µg/L. Ammonium concentrations in mg/L. *The MFC1 borehole was not nested and contained both anode and cathode

TPH CWG & ammonium	MFC3 anode		MFC3 cathode		MFC2 anode		MFC2 cathode		MFC1*	
	Prior	After	Prior	After	Prior	After	Prior	After	Prior	After
Ammonium	2.5	0.40	1.0	0.087	32	7.7	4.9	0.58	0.23	< 0.050
Aliphatic > C5-C6	< 0.10	< 0.10	< 0.10	< 0.10	< 0.10	< 0.10	< 0.10	< 0.10	< 0.10	< 0.10
Aliphatic > C6-C8	< 0.10	< 0.10	< 0.10	< 0.10	< 0.10	< 0.10	< 0.10	< 0.10	< 0.10	< 0.10
Aliphatic > C8-C10	< 0.10	1500	< 0.10	< 0.10	65	1600	< 0.10	< 0.10	< 0.10	< 0.10
Aliphatic > C10-C12	< 0.10	120	< 0.10	< 0.10	52	720	< 0.10	69	< 0.10	< 0.10
Aliphatic > C12-C16	< 0.10	610	< 0.10	< 0.10	< 0.10	550	< 0.10	400	< 0.10	< 0.10
Aliphatic > C16-C21	< 0.10	< 0.10	< 0.10	< 0.10	< 0.10	< 0.10	< 0.10	770	< 0.10	< 0.10
Aliphatic > C21-C35	< 0.10	< 0.10	< 0.10	< 0.10	< 0.10	< 0.10	< 0.10	760	< 0.10	< 0.10
Aliphatic > C35-C44	< 0.10	< 0.10	< 0.10	< 0.10	< 0.10	< 0.10	< 0.10	< 0.10	< 0.10	< 0.10
Total aliphatic hydrocarbons	< 5.0	2200	< 5.0	< 5.0	120	2900	< 5.0	2000	< 5.0	< 5.0
Aromatic > C5-C7	520	7800	< 0.10	< 0.10	3600	2600	< 0.10	< 0.10	< 0.10	< 0.10
Aromatic > C7-C8	73	2400	< 0.10	< 0.10	4700	3500	< 0.10	< 0.10	< 0.10	< 0.10
Aromatic > C8-C10	270	2000	91	170	7800	3000	< 0.10	< 0.10	< 0.10	< 0.10
Aromatic > C10-C12	220	2400	180	190	10000	2100	< 0.10	< 0.10	< 0.10	< 0.10
Aromatic > C12-C16	61	700	300	290	2200	700	< 0.10	89	< 0.10	< 0.10
Aromatic > C16-C21	< 0.10	< 0.10	9.6	< 0.10	250	< 0.10	< 0.10	34	< 0.10	< 0.10
Aromatic > C21-C35	< 0.10	< 0.10	< 0.10	< 0.10	< 0.10	< 0.10	< 0.10	150	< 0.10	< 0.10
Aromatic > C35-C44	< 0.10	< 0.10	< 0.10	< 0.10	< 0.10	< 0.10	< 0.10	< 0.10	< 0.10	< 0.10
Total aromatic hydrocarbons	1100	15,000	580	650	29,000	12,000	< 5.0	270	< 5.0	< 5.0
Total petroleum hydrocarbons	1100	17,000	580	650	29,000	15,000	< 10	2300	< 10	< 10

MFC 1 recorded aromatic and aliphatic hydrocarbon concentrations that fell below the detection limits, corresponding to its relatively low average current production of approximately 1.35 µA during the 10 kOhms operation. On the other hand, MFC 2 exhibited substantial reductions in all aromatic fractions and TPH, although its average current production, approximately 0.99 µA at 10 kOhms operation, was less than that of MFC 1. Notably, MFC 2, situated at the plume center, initially had the highest concentration of 29,000 µg/L, which decreased to 15,000 µg/L by the end of the operational period. This decline suggests that despite significant fluctuations in contamination levels, they remained too elevated for microbial colonization within the MFC. Overall, the results highlight that MFCs located between the deep plume center with high organic matter and the oxygen-rich layers above failed to generate significant or sustained power output consistently. In the case of MFC 3 (plume fringe), an increase in TPH was observed after the monitoring period, potentially related to excessive borehole purging that introduced additional contamination from the plume before sampling. However, MFC 3 demonstrated higher current production (~ 21 µA) throughout the monitoring periods, suggesting ongoing microbial activity at the MFC.

### Electrode surface characterization

The surface area analysis did not reveal any distinctions between the original GAC and the GAC utilized at the study site (Supplementary Table S2). The movement of groundwater through the electrodes did not induce alterations in the physical properties of the GAC. The recorded values align with published data, indicating that GAC’s micro-porosity results in a substantial specific surface area ranging from 2 to 540 m^2^/g, significantly surpassing the geometric surface of large granules (Lehmann and Joseph 2012). Alterations of the surface area of anode electrodes primarily stem from sorption processes, while alterations in the surface area of cathode electrodes are associated with precipitation. Removing contaminants in microbial fuel cells is influenced by sorption and oxidation at the anode and precipitation and reduction at the cathode electrodes (Alkhadra et al. 2022; Mao et al. 2023; Mohanakrishna et al. 2018; Mosquera-Romero et al. 2023; Yuan et al. 2022).

Interestingly, despite MFC 2 being more contaminated, it displayed a slightly lower surface area impact (754.42 m^2^/g) than MFC 3 (739.24 m^2^/g). The diminished surface area observed in MFC 3 might indicate heightened microbial growth or biofilm formation at the electrode surface. The XRD analysis of the GAC unveiled that quartz and calcite are the predominant crystalline minerals in each sample. While quantitative interpretation remains challenging with XRD data, it is evident that there is a noticeable alteration in the crystalline phase compared to the original sample. The shifts in the peaks relative to this sample signify an increase in grain size, affirming the occurrence of adsorption during the experimental period.

Principal component analysis (PCA) was used to analyze the raw XRD data within the 2θ range of 10–50°. To ensure consistency, all XRD data points were normalized using a centered log ratio, and the PCA analysis was executed using R software. The resulting PCA is graphically represented along two axes (Supplementary Fig. S5). The y-axis (PCA1) reflects the quantity of available surface area, which correlates with the BET measurements. Consequently, the greater the available pore space within the GAC, the more likely it is for the PCA1 value to exhibit a positive trend. This ranking of pore space availability from highest to lowest goes as follows: GAC untreated > MFC 2 cathode > MFC 3 anode > MFC E cathode > MFC 1 anode > MFC 1 cathode > MFC 2 anode. It is important to mention that certain BET measurements may not perfectly align with this sequence, potentially due to the chance of overestimating the surface area of GAC, attributed to the heightened adsorption occurring in micropores (Paulino et al. 2023). PCA2 positioned along the x-axis is likely associated with the presence of aerobic conditions (related to precipitation or inorganic material at cathodes) and anaerobic conditions (associated with sorption of organic matter or microbial material) within each borehole.

## Discussion

Traditionally, it was conventionally believed that groundwater plumes exhibited distinct zones of electron acceptors, forming a “thermodynamic ladder.” This concept posited a sequence starting with aerobic processes at the plume’s periphery, followed by nitrate reduction, iron (III) oxide reduction, manganese (IV) reduction, sulfate reduction, and culminating in methanogenic processes at the plume’s central region (Lovley et al. 1994). However, recent research has contested this assumption, demonstrating that various respiration processes, including methanogenesis, iron and manganese reduction, may concurrently take place over relatively small spatial areas within a groundwater plume (Casiraghi et al. 2022; Kirmizakis 2020; Rhea and Clark 2022). It is now suggested that the microbial architecture and distribution in the subsurface is influenced not only by thermodynamic factors but also by ecological, physiological, and mutualistic factors (Bethke et al. 2011). Consequently, the concept of distinct longitudinal zones of electron acceptors within the plume has been replaced by a thin clustered zone of activity at the plume fringe (Meckenstock et al. 2015). This shift in understanding has significant implications for monitoring and engineering effective remediation solutions for groundwater plumes. Here, we tested a hypothesis that an MFC would only function well at the plume fringe, with a diverse ecological niche of degraders and electrogens proliferating.

Based on the groundwater chemistry within the boreholes, the site’s southern section contains the primary portion of the contaminated plume at depth. The plume can be simply delineated by groundwater redox but has the chemical characteristics typical of a carbureted water gas (CWG) process dominated by lighter aromatic fractions (Gallacher et al. 2017). Previous microbial and viral analyses (Costeira et al. 2019) have provided evidence of a diverse microbial community surrounding the plume associated with hydrocarbon-contaminated environments. Chemometric analysis of the ratio of BTEX compounds in deep sandstone aquifers also suggested that degradation was happening within the plume but could not differentiate whether this was due to physical transport (sorption, dilution) or due to microbial activity. Based on the redox and BTEX chemistry, MFC locations were selected within the northern sector of the site as control the plume center and the plume fringe as MFC locations with the anode in the sandstone at depth and the cathode in shallow alluvial overburden.

**Fig. 5:**
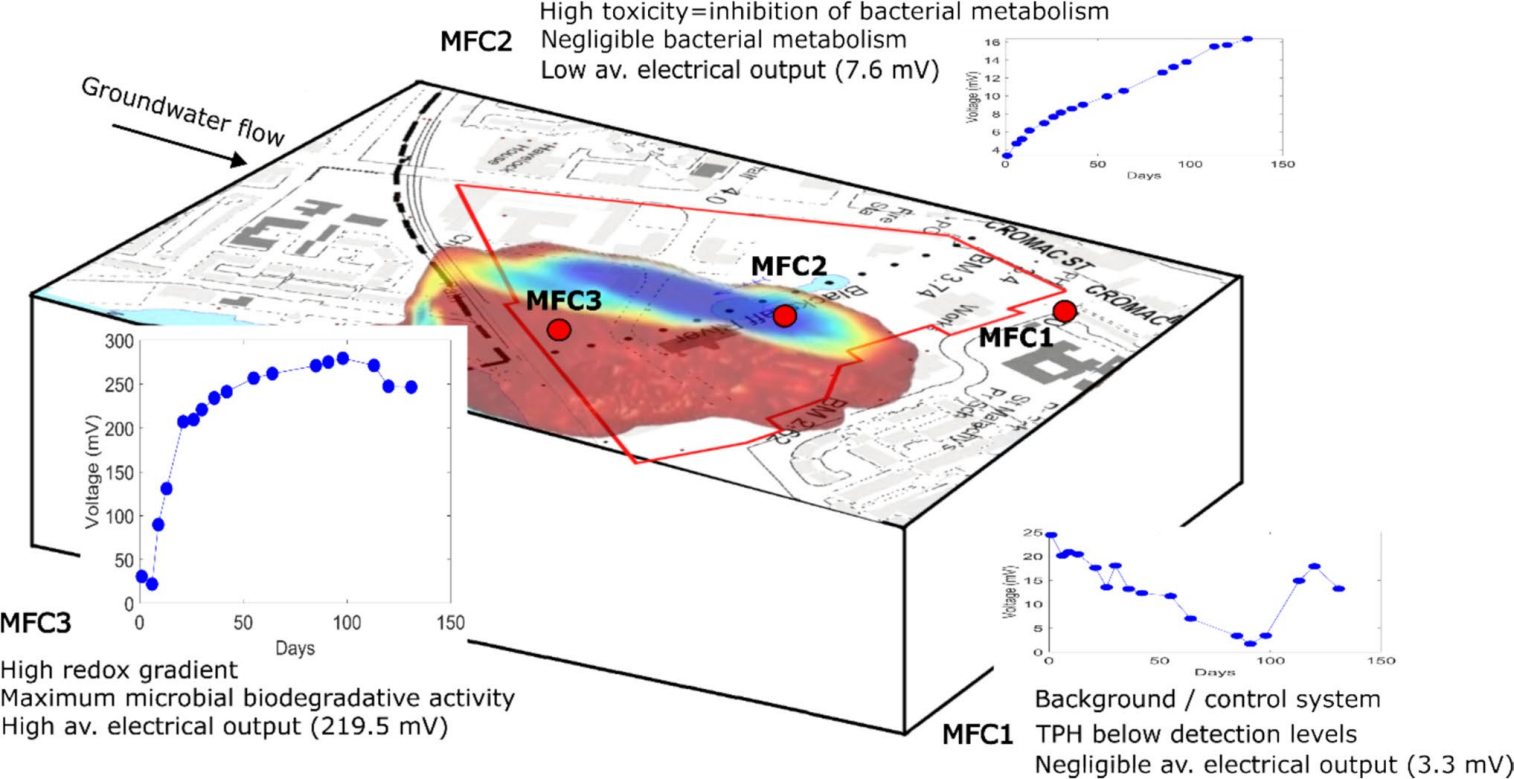
Conceptual site model of groundwater plume with locations of the control (MFC1), the plume center (MFC2), and the plume fringe (MFC3)

The electrical monitoring provided a deeper understanding of the chosen sites and the significance of a good conceptual site model prior to installation. MFC 1 (control) showed negligible voltage as the electrodes did not develop microbial growth during the monitoring period (Fig. 5). MFC 2 (plume center) also showed little electrical output and faced challenges due to the high contamination, which was likely toxic to microbial activity (Ferguson et al. 2007, 2003). In addition, this lack of electrical signal from the electrodes at the plume center also suggests that the MFC electrodes are not acting as an abiotic proxy for redox conditions. Improved performance for MFC 2 could be achieved through amended conditions, such as coupling with other more active remediation strategies to accelerate biodegradation.

On the other hand, MFC 3, located at the plume fringe, exhibited a continuous electrical output due to an abundance of electrogenic bacteria and degraders. This aligns with the expected behavior plume at a plume fringe. The anode of MFC 3 was in a contaminated environment, and microbial analysis highlighted the presence of key electrogenic taxa that produce and transfer electrons such as *Desulfuromadaceae* and *Geobacter* which aligns with the electrical data. These electrons were utilized at the cathode potentially by aerobic degraders and though precipitation as highlighted by analysis of the GAC by BET and XRD. The principal objective of these microbial fuel cells (MFCs) was not electricity generation but rather to pinpoint the position of the plume’s fringe and evaluate whether its microbial ecosystem supports degradation. This straightforward method of examining the microbial ecology across extensive electrodes within contaminant plumes can provide insights for decisions related to monitoring, improving, or formulating remediation strategies.

While it is not always possible to establish a simple linear relationship between contaminant concentration and current output in MFCs, this study illustrates the utility of MFCs for contamination monitoring. MFCs are affected by complex factors such as microbial activity, substrate availability, and contaminant types, often resulting in non-linear dynamics where contaminant concentration does not directly correspond to current output. For example, in MFC 2, high contamination levels likely inhibited microbial activity, while in MFC 3, electrogenic bacteria at the plume fringe maintained consistent electrical output. Despite these non-linearities, MFCs remain valuable for real-time monitoring, as they produce electrical signals responding to contaminant degradation. Furthermore, their self-powered nature makes them a sustainable option for long-term environmental monitoring. The relationship between pollutant concentration and current output may not be linear, but MFCs can still detect changes and trends in pollutant levels. In this context, the continuous electrical output from MFC 3 at the plume fringe offers valuable data on microbial activity and degradation potential. This real-time insight into microbial dynamics can support early detection of environmental contamination and inform remediation strategies. Therefore, even without a linear relationship, MFCs are highly effective tools for understanding pollution dynamics and supporting environmental monitoring.

The significant outcomes of this study highlight its novelty compared to other similar studies. This research presents an economically efficient and straightforward design for MFCs that can be easily integrated into existing boreholes to monitor NA around groundwater plumes. Unlike many MFC studies, which are typically conducted in controlled lab environments or focus on short-term remediation strategies, this study demonstrates a field-scale implementation using long electrodes across redox boundaries in contaminated sites, specifically a former gasworks facility. The MFCs, especially the one placed at the plume fringe (MFC 3), exhibited sustained electrical output, demonstrating a strong correlation between microbial activity and natural degradation processes.

## Conclusion

This study functions as a proof of concept, demonstrating the successful colonization and electronic utilization of GAC electrodes by diverse microbial communities within contaminated groundwater plumes. The ability to add electrodes easily and affordably to existing boreholes makes this method attractive for continuously tracking microbial populations in real time. A novel aspect of the study is the use of MFCs as real-time biosensors for microbial activity related to natural attenuation, offering a continuous monitoring solution that is self-powered and sustainable. The study also underscores the importance of the plume fringe in microbial degradation, providing valuable insights for designing and improving remediation strategies. This approach contrasts with typical remediation methods that often rely on active interventions, demonstrating the potential for MFCs to be used in passive, long-term environmental monitoring without requiring external power sources. The MFC shows promise as an eco-friendly remediation solution, suitable for both MNA and enhanced remediation processes. Its adaptability also extends to other contaminated sites, where electrogenic microbial communities can flourish independently without the need for additional substrates. However, while MFCs are highly effective in certain environments, their performance may be influenced by the type of contaminants present. Contaminants such as heavy metals and chlorinated solvents can be toxic to microbial communities, potentially inhibiting microbial growth and electron transfer, which could reduce MFC efficiency in monitoring or remediation efforts. Additionally, the durability of MFC materials, such as electrodes, may be compromised in harsh environments, leading to maintenance challenges over time. It is also important to mention that although these systems were not originally designed to generate significant electrical power due to factors like internal resistance, this does not diminish their potential as valuable tools for monitoring biodegradation. Despite large-scale MFC not being efficient for energy production, they remain practical, user-friendly, and sustainable remediation tools. Their ability to monitor diverse microbial activities in real-time across various contaminants and environmental conditions makes them a versatile solution with broad applications in environmental monitoring. Further research into how different contaminants affect MFC performance and microbial community structure will help refine these systems for use in a wider range of contaminated sites.

## Supplementary Information

Below is the link to the electronic supplementary material.

Supplementary file1 (DOCX 1154 KB)

## Data Availability

Data is available in the supplementary information and the raw 16S rRNA gene sequences are available at the European National Archive (ENA) associated with the study accession ID PRJEB64905, as fastq files https://www.ebi.ac.uk/ena/browser/view/PRJEB64905.
