# Supplementary material for: Microbial fuel cells to monitor natural attenuation around groundwater plumes
Source: Environ Sci Pollut Res Int. 2025 Jan 4;32(4):2069–84. doi: 10.1007/s11356-024-35848-5 (PMC11775044; doi:10.1007/s11356-024-35848-5)
Supplement: Supplementary file 1 — Supplementary file1 (DOCX 1154 KB) [file 11356_2024_35848_MOESM1_ESM.docx]

Supplementary Material

**Bio-Electrochemical Systems to Monitor Natural Attenuation around Groundwater Plumes**

P. Kirmizakis^1^, M. Cunningham^2^, D. Kumaresan^2^, R. Doherty^3^,

^1^ Department of Geosciences, College of Petroleum Engineering and Geosciences, King Fahd University of Petroleum and Minerals, Dhahran, Saudi Arabia

^2^ School of Biological Sciences, Queen's University Belfast, UK

^3^ School of Natural and Built Environment, Queen's University Belfast, UK

**Figure S1.** Design of BES electrodes placed on site


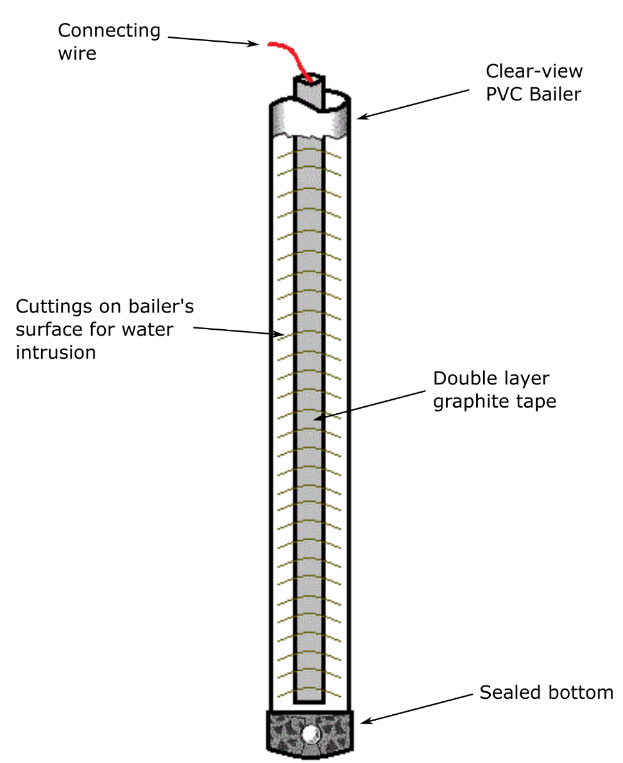


**Table S1** Measured retardation factors for BTEX compounds of overburden and sandstone aquifers

|  | R1 = (B+T)/(E+X) | R2 = B/T | R3 = B/X | R4 = T/E | R5 = X/E |
| --- | --- | --- | --- | --- | --- |
| Overburden (unconfined) aquifer | | | | | |
| B01 | 0.125 | 1 | 0.17 | 0.1 | 0.6 |
| B02 | 0.66 | 1 | 0.5 | 1 | 2 |
| B04 | 0.66 | 1 | 0.5 | 1 | 2 |
| B05 | 5.66 | 0.06 | 0.5 | 16 | 2 |
| B06 | 0.66 | 1 | 0.5 | 1 | 2 |
| B07 | 2.31 | 17 | 5.48 | 0.21 | 0.66 |
| B09 | 0.78 | 7.5 | 1 | 0.30 | 2.22 |
| BH101 | 0.66 | 1 | 0.5 | 1 | 2 |
| BH102 | 0.66 | 1 | 0.5 | 1 | 2 |
| WS200 | 0.66 | 1 | 0.5 | 1 | 2 |
| WS202 | 0.66 | 1 | 0.5 | 1 | 2 |
| WS206 | 5.59 | 18 | 7.83 | 0.91 | 2.09 |
| DG02 | 0.66 | 1 | 0.5 | 1 | 2 |
| Sandstone (confined) aquifer | | | | | |
| SS1 | 1 | 1 | 0.5 | 1 | 2 |
| SS2 | 491.6 | 1272.7 | 595.7 | 2.2 | 4.7 |
| SS4 | 4.9 | 0.91 | 2.6 | 0.1 | 9.5 |

Table S2 BET Surface Area of GAC samples.

| GAC sample | Surface Area |
| --- | --- |
| GAC - Untreated | 766.9098 m^2^/g |
| GAC – MFC 1 Anode (SS1) | 770.2175 m^2^/g |
| GAC – BES 1 Cathode (OB1) | 733.4873 m^2^/g |
| GAC – BES 2 Anode (SS4) | 754.4243 m^2^/g |
| GAC – BES 2 Cathode (OB4) | 758.5940 m^2^/g |
| GAC – BES 3 Anode (BH3.D) | 739.2385 m^2^/g |
| GAC – BES 3 Cathode (BH3.S) | 751.5286 m^2^/g |

**Figure S2**  Distribution patterns of bacterial genus in the samples from microbial fuel cell anode and cathode electrodes. MFC3 = high electrical output, MFC2 = low electrical output. Labelling notation MFC3AR1 = MFC3, Anode, Replicate 1


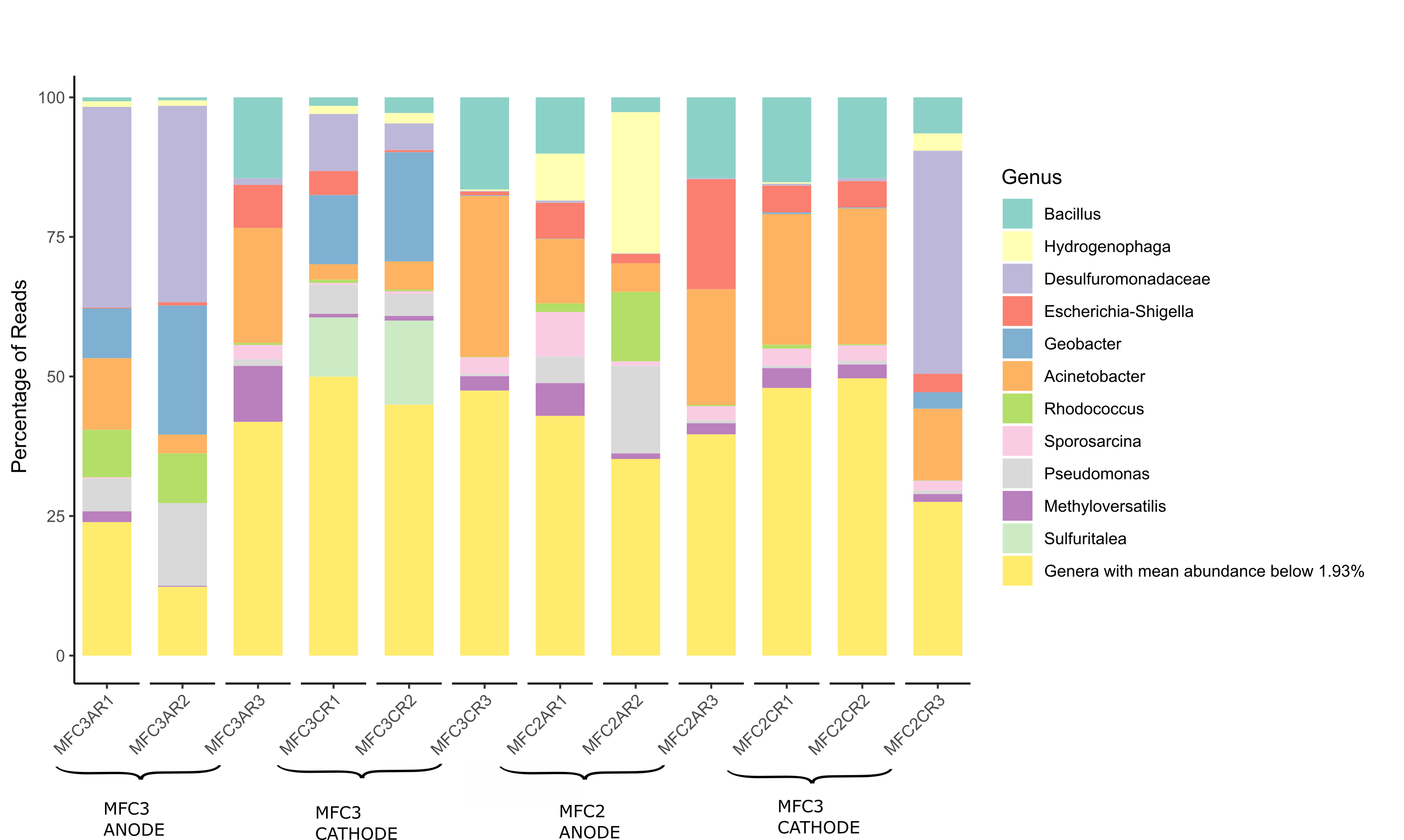


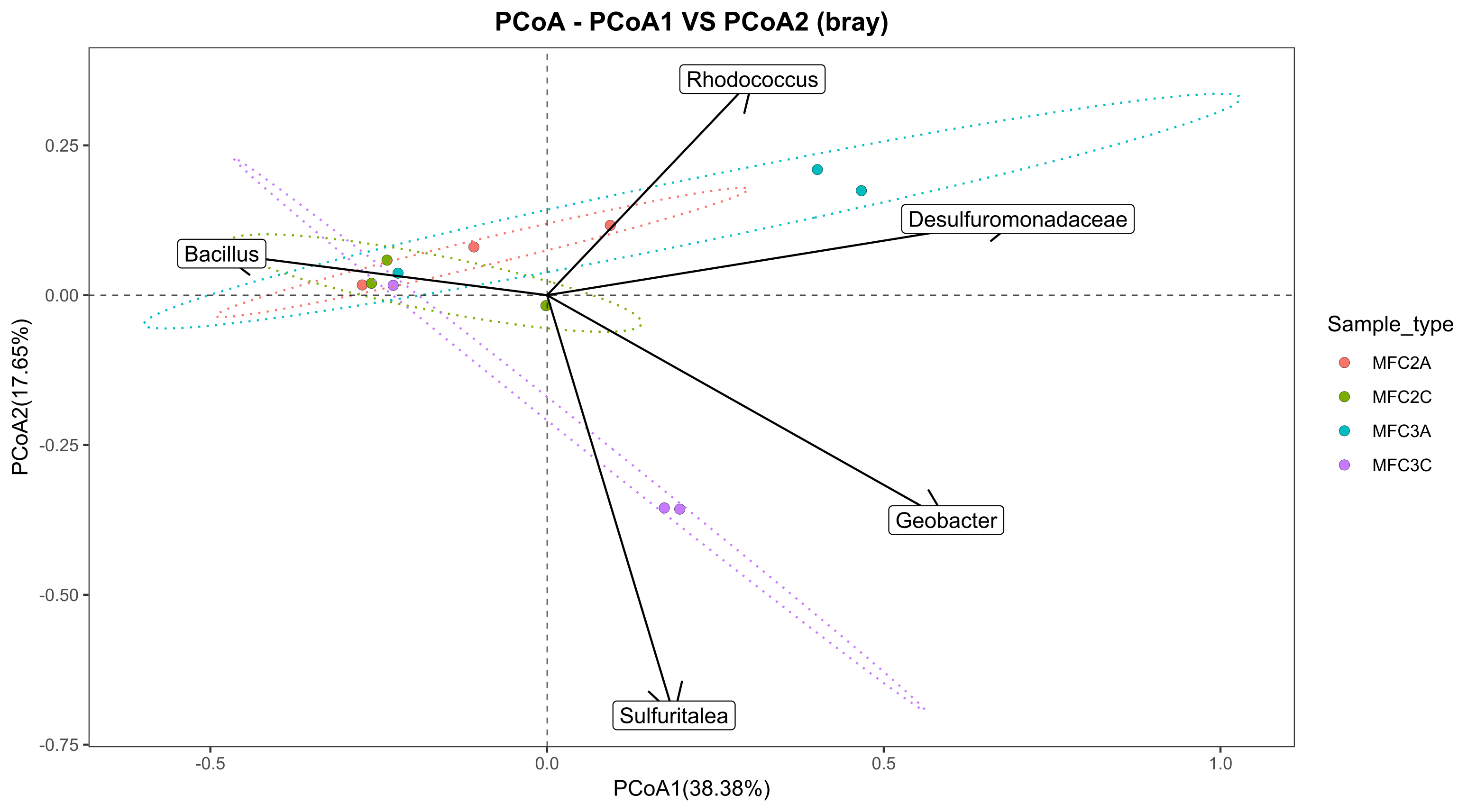


**Figure S3** Beta diversity Bray-Curtis plot


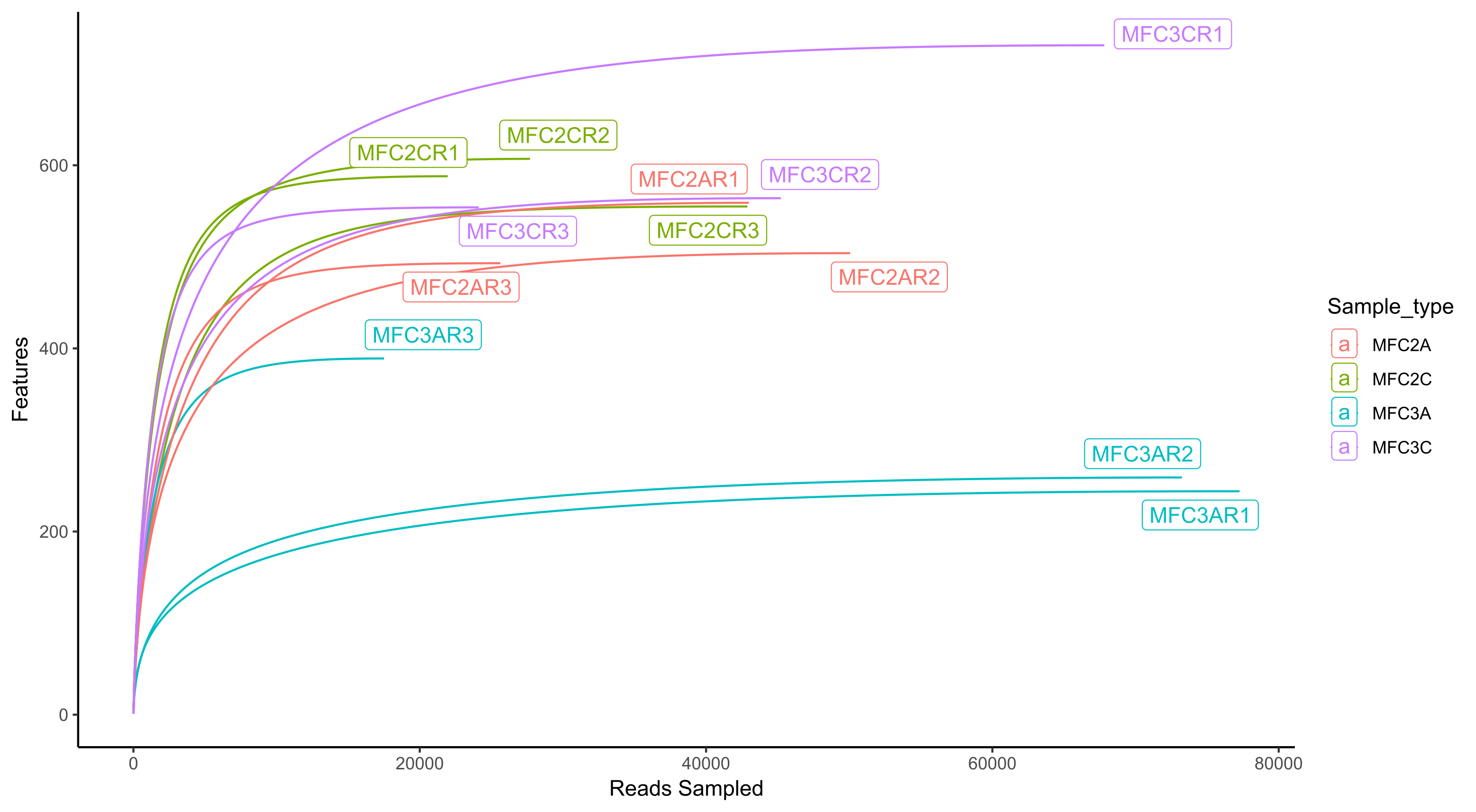


**Figure S4 .** Rarefaction curve after QIIME2 analysis prior to filtering samples using phyloseq.


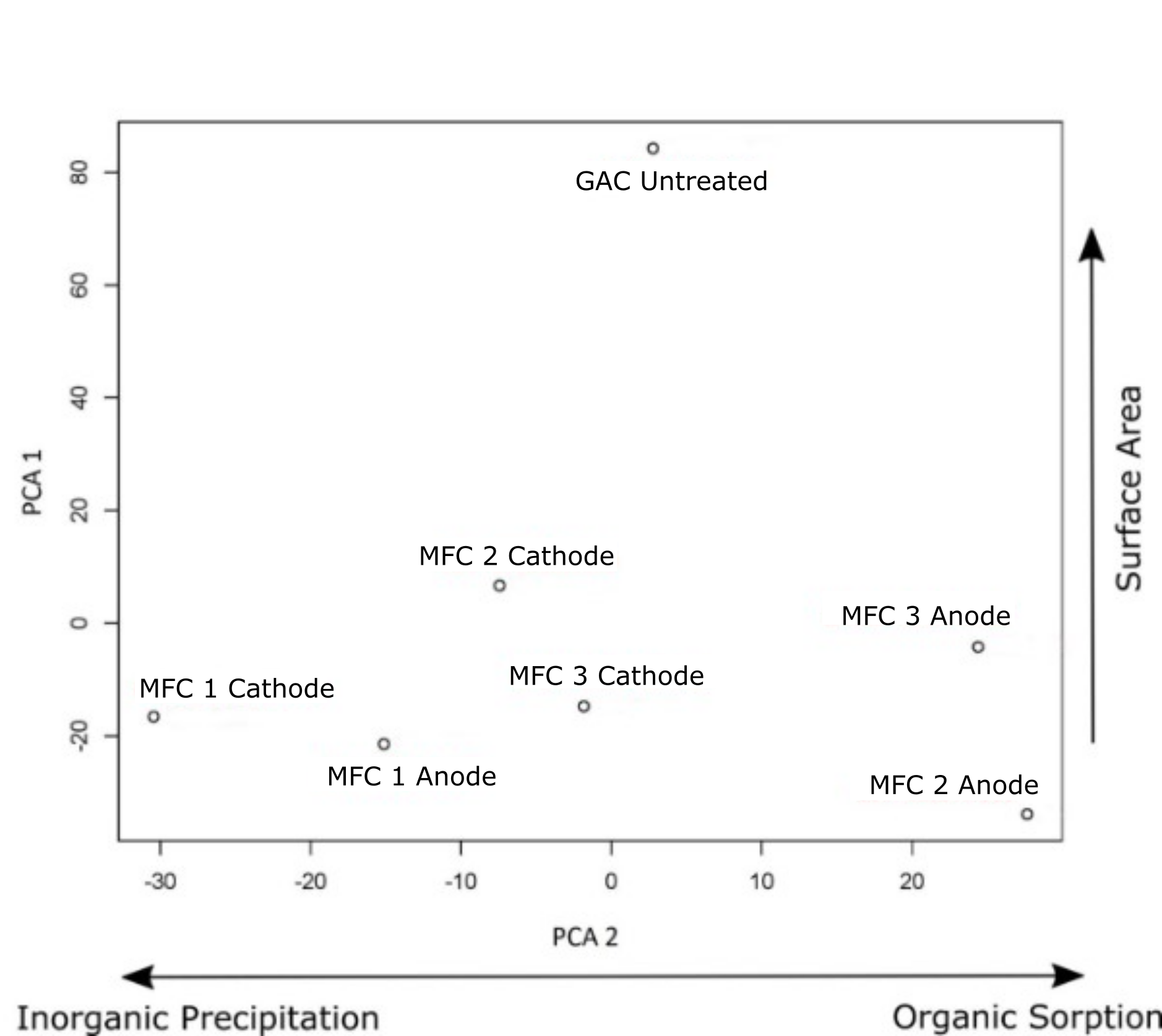


**Figure S5** Principal Component Analysis of XRD analysis of the electrodes post monitoring, PC1 (y-axis) is dominated by surface area or the amount of precipitation, PC2 (x-axis) is dominated the sorption / precipitation mechanisms on the electrode.
